# Applying multinomial logistic regression to prediction effect of environmental and vehicle type factors on the severity of road accidents (Case study: Tabriz-Ahar Road)

**Published:** 2019-08

**Authors:** Yousef Kazempour, Saeed Givehchi, Hassan Hoveidi

**Affiliations:** ^*a*^Faculty of Environment, University of Tehran, Tehran, Iran.

## Abstract

**Background:**

Determining the factors that have an important role in increasing the severity of accidents is important to reduce or eliminate mortality and severity of injuries caused by accidents. According to the World Health Organization, the mortality rate in Iran is 32.1 per 100,000 population. Which is much higher than the population of 8.7 per 100,000 people, compared with other high-income countries.

**Methods:**

In this study road variables, environmental and climate variables, and vehicle types for four years (1394-1391) were selected accident data of Tabriz-Ahar road for review. The severity of crashes was classified into three levels include fatal, injured and PDO. In this study, multinomial logistic regression method was used to determine the probability and predict the severity of accidents.

**Results:**

The results showed that in the case of injured crashes compared with PDO, rainy weather (OR = 0.028), intersections (OR = 0.044) increase the severity of accidents. Also, in the case of fatal accidents compare with PDO, Driving during the night (OR = 0.005), intersections (OR = 2.24) and increase in heavy vehicles on the road (OR = 4.31) increase the severity of fatal accidents.

**Conclusions:**

The results of this study show that the multinomial logistic regression model offers a promising approach to predict the severity of accidents in future studies. According to the results, all the significant factors mentioned above should be improved. Therefore, it is necessary to reduce the severity of accidents by taking measures such as development Grade separation intersections at black spots and also increasing the number of lane and separating the slow lane from overtaking lane on this road.

**Keywords:**

Crash severity, Logistic Regression, Road Safety, Crash modelling

